# Is there a common latent cognitive construct for dementia estimation across two Chinese cohorts?

**DOI:** 10.1002/dad2.12356

**Published:** 2022-09-14

**Authors:** Yuyang Liu, Yanjuan Wu, Jingheng Cai, Yun Huang, Yuntao Chen, Tishya M. Venkatraman, Sophia Lobanov‐Rostovsky, Piotr Bandosz, Yung‐Jen Yang, Yu‐Tzu Wu, Jing Liao, Yuantao Hao, Eric J. Brunner

**Affiliations:** ^1^ Department of Medical Statistics School of Public Health Sun Yat‐sen University Guangzhou People's Republic of China; ^2^ Sun Yat‐sen Global Health Institute School of Public Health and Institute of State Governance Sun Yat‐sen University Guangzhou People's Republic of China; ^3^ Department of Statistics Sun Yat‐sen University Guangzhou People's Republic of China; ^4^ Guangdong Provincial Center for Disease Control and Prevention Guangzhou People's Republic of China; ^5^ Department of Epidemiology & Public Health University College London London UK; ^6^ Department of Public Health and Policy University of Liverpool Liverpool UK; ^7^ Department of Prevention and Medical Education Medical University of Gdansk Gdansk Poland; ^8^ Social Research Institute Institute of Education University College London London UK; ^9^ Population Health Sciences Institute Newcastle University Newcastle UK; ^10^ Peking University Center for Public Health and Epidemic Preparedness & Response Beijing People's Republic of China

**Keywords:** China, cognitive impairment, confirmatory factor analysis, dementia, epidemiology

## Abstract

**Introduction:**

It is valuable to identify common latent cognitive constructs for dementia prevalence estimation across Chinese aging cohorts.

**Methods:**

Based on cognitive measures of 12015 Chinese Longitudinal Healthy Longevity Survey (CLHLS; 13 items) and 6623 China Health and Retirement Longitudinal Study (CHARLS; 9 items) participants aged 65 to 99 in 2018, confirmatory factor analysis was applied to identify latent cognitive constructs, and to estimate dementia prevalence compared to Mini‐Mental State Examination (MMSE) and nationwide estimates of the literature.

**Results:**

A common three‐factor cognitive construct of orientation, memory, and executive function and language was found for both cohorts with adequate model fits. Crude dementia prevalence estimated by factor scores was similar to MMSE in CLHLS, and was more reliable in CHARLS. Age‐standardized dementia estimates of CLHLS were lower than CHARLS among those aged 70+, which were close to the nationwide prevalence reported by the COAST study and Global Burden of Disease.

**Discussion:**

We verified common three‐factor cognitive constructs for both cohorts, providing an approach to estimate dementia prevalence at the national level.

**Highlights:**

- Common three‐factor cognitive constructs were identified in Chinese Longitudinal Healthy Longevity Survey (CLHLS) and China Health and Retirement Longitudinal Study (CHARLS).
- Crude dementia estimates using factor scores were reliable in both cohorts.
- Estimates of CHARLS were close to current evidence, but higher than that of CLHLS.

## 1 BACKGROUND

Dementia is a clinical neurodegenerative syndrome characterized by progressive loss of cognitive function and ability to perform daily activities.^1^ It negatively impacts patients’ and their families’ quality of life, and leads to substantial health and social care costs.^2^ Evidence has been accumulating on the prevalence of dementia in China.^3^, ^4^, ^5^ Yet, there is limited evidence on the nationwide estimates and existing epidemiological surveys generally focus on specific regions that might not be representative of the older population across the whole country.^6^

Two aging cohorts, Chinese Longitudinal Healthy Longevity Survey (CLHLS)^7^ and China Health and Retirement Longitudinal Study (CHARLS)^8^ have been established to provide information on social, economic, and health circumstances of older Chinese adults. Clinical diagnosis of dementia is not available, but multiple measures of cognitive and functional abilities have been collected. These data provide an opportunity to explore a novel approach to generate nationwide estimates for the prevalence of dementia.

To estimate the nationwide prevalence of dementia using existing data, one of the key challenges is variation in the cognitive measures used across cohorts. There are several widely applied approaches in calibrating cognitive items across studies.^9^ One method is to use confirmatory factor analysis (CFA), which assumes that although the set of cognitive tests differs, the underlying common latent cognitive function construct can be derived.^9^, ^10^ CFA has been applied to facilitate calibrating cognitive performance,^9^ ^—^ ^11^ physical functioning,^12^ and mental states^13^ across datasets. To achieve multi‐cohort comparison, the first step is to confirm that the same psychometric structure can be established across cohorts. If the observed factor structure is similar across different studies and model fit statistics are adequate in each study, then further analysis of the psychometric equivalence of a construct across groups or across time would be possible.^14^

This study aimed to identify common latent cognitive constructs for dementia prevalence estimation across these two population‐representative Chinese aging cohorts. We first characterized the extent to which cognitive measures administered in the two cohorts can be considered to measure similar latent cognitive construct, and then compared how the dementia prevalence estimated by our latent approach corresponded to established cognitive function measures and the prevalence estimates reported in the literature.

## 2 METHODS

### 2.1 Study samples

**FIGURE 1:**
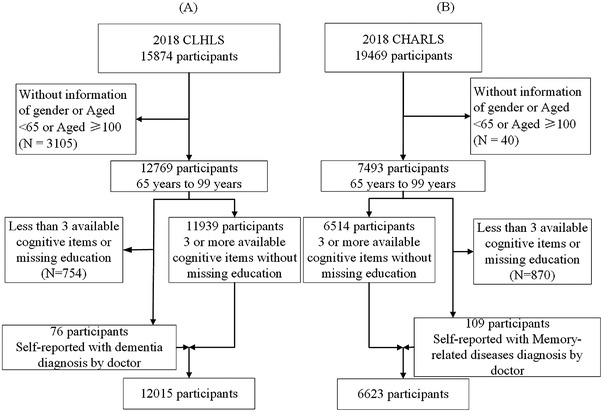
Sample selection flowcharts for the CLHLS and CHARLS 2018 wave samples. CHARLS, China Health and Retirement Longitudinal Study; CLHLS, Chinese Longitudinal Healthy Longevity Survey

CLHLS and CHARLS are ongoing nationwide surveys on health and its risk factors of older adults, with participants aged 65+ years for CLHLS and 45+ years for CHARLS. The first wave of CLHLS and CHARLS was 1998 and 2011, respectively, by using household surveys, with a follow‐up interval of 2 to 3 years. The latest wave for both cohorts was conducted in 2018. The present study included participants aged 65 to 99 in CLHLS (*n* = 12015) and CHARLS (*n* = 6623) in the latest wave. Participants were included if they had at least three cognitive measures available, or reported any doctor‐diagnosed dementia or memory‐related diseases. Figure 1 shows the sample selection flowchart. Written informed consent was obtained from all study participants.

**RESEARCH IN CONTEXT**

1. **Systematic Review**: The authors reviewed the literature using traditional (e.g., PubMed) sources. To calibrate cognitive items across studies, one method is to use confirmatory factor analysis (CFA), which has been applied to calibrate cognitive performance, physical functioning, and mental states across datasets. These relevant citations are appropriately cited.
2. **Interpretation**: Based on CFA, our study identified similar cognitive constructs of two population‐representative Chinese aging cohorts. Configural invariance established in this study facilitated the calibration of different cognitive measures across the two cohorts, and generation of nationwide estimates for the prevalence of dementia in China.
3. **Future Directions**: We verified common three‐factor cognitive constructs for both cohorts, providing an approach to estimate dementia prevalence at the national level.

### 2.2 Assessment of cognitive function in CLHLS and CHARLS

CLHLS used the modified version of the Chinese Mini‐Mental State Examination (C‐MMSE) by reducing items associated with literacy.^7^ Specifically, four items that were considered less closely related to the individual daily life were deleted (i.e., the name of province/city, county/district, and township/town/street, as well as the number of the floor), and the item on “say a complete sentence” was replaced by “name different kinds of food in 1 minute.”^15^ Altogether, 13 cognitive items remained with a summary score range from 0 to 30.^16^ According to the psychometric structure of MMSE,^16^, ^17^ these items assessed orientation of time (i.e., season, month, time of day, date of mid‐autumn festival) and location (i.e., name of county), memory (i.e., immediate and delayed recall of three words),^16^ as well as executive function and language (i.e., repeating a sentence, naming objects and food, hand‐fold‐leg test, subtraction, and drawing).^1^, ^16^, ^17^, ^18^

CHARLS administrated the telephone interview for cognitive status questionnaire (TICS) via face‐to‐face interview.^19^ As a modified version of MMSE, TICS examined similar cognitive domains with 9 items but similar summary score range (range 0–31), namely five tests for orientation to time (i.e., season, month, day, year, and day of the week),^19^ two tests for memory (i.e., immediate and delayed recall of 10 words^18^), and two tests for executive function and language (i.e., number subtraction and drawing).^16^, ^19^ The 2018 wave of CHARLS also included C‐MMSE (range 0–30),^20^ without modification to literacy as CLHLS.^15^ In the current analysis, the TICS was used to build the CFA model, while the C‐MMSE was used for method comparison.

### 2.3 Assessment of physical function in CLHLS and CHARLS

Physical function in both cohorts was assessed by the basic activities of daily living (ADL), including (1) getting in or out of bed, (2) bathing, (3) dressing, (4) cutting food and eating, (5) using the toilet, and (6) controlling urination and bowel movement. Despite using the same ADL items, answer options slightly differed: three options regarding needs for assistance were used in CLHLS, while four options regarding difficulty and needs for help were used in CHARLS (see details in Table S1 in supporting information). To make these options comparable, participants who needed assistance (CLHLS) or help (CHARLS) in daily activities were considered dependent.

### 2.4 Case definition of dementia

We adapted the dementia case definition to resemble Diagnostic and Statistical Manual of Mental Disorders (DSM)‐IV, DSM‐5, and International Classification of Diseases 10th edition for diagnosis of dementia whereby measures of cognitive impairment, functional impairment, and adjudicated dementia diagnosis available in the data were mapped onto the key elements of these criteria (see details about comparison to DSM‐IV in Table S2 in supporting information). Cognitive impairment was defined as an impairment in two or more domains of cognitive function. Domain‐specific impairment was quantified as a score of 1.5 standard deviations below the mean^21^ compared to the population aged 65 to 99 years with the same level of education, categorized into three levels: 0 years; 1 to 6 years, and 7 years or higher. Those who were dependent on performing one or more basic ADLs were defined as functionally impaired.^22^ Dementia was defined as those with a combination of cognitive and functional impairment, or those who had self‐reported doctor‐diagnosed dementia or memory‐related diseases.

### 2.5 Statistical analysis

Confirmatory factor analysis was used to identify latent cognitive function structure across the two cohorts.^23^ This method performed well in identifying multi‐factor structure and could accommodate cognitive measures with skewed distribution.^9^ To verify whether a similar factor structure was derived from both cohorts, parallel analysis, a method in exploratory factor analysis (EFA), was used to determine the number of latent factors suitable for each cohort.^24^ In the parallel analysis, eigenvalues from the real data set were compared to those simulated from 50 random data sets generated by the Monte Carlo simulation technique, with the 95th percentile criterion.^25^ The number of factors retained was determined by observed eigenvalues falling above the 95^th^ percentile of the random eigenvalues.

The multifactor CFA model was then built for CLHLS and CHARLS separately based on all available cognitive measures, the number of factors indicated by EFA, and the conceptual framework of potential cognitive domains suggested by the cognitive literature,^16^, ^19^ DSM‐IV‐TR,^26^ and DSM‐5.^18^ To establish the configural invariance of the two cohorts,^27^ CLHLS, having more cognitive items than CHARLS, was chosen as the reference cohort. Mean and variance of its common factor orientation was set to 0 and 1, respectively. CFA was carried out first for CLHLS. We then performed CFA for CHARLS, with the threshold and factor loading of the shared cognitive item (month) constrained to be equivalent across cohorts. All other non‐fixed parameters were freely estimated. Due to cognitive tests with skewed distributions, all tests were treated as ordinal categorical variables.^9^ The weighted least squares mean and variance adjusted (WLSMV) estimators and a polychoric correlation matrix of variance were used.^28^ A factor loading higher than 0.60 was acceptable.^29^ Model fit was assessed by the root mean square error of approximation (RMSEA), standardized root mean square residual (SRMR), and comparative fit index (CFI). Values of RMSEA < 0.06, SRMR < 0.08, and CFI > 0.95 indicate good model fit.^30^

Once adequate model fit was verified for the given cohort, factor scores of each identified cognitive domain were derived from these multifactor CFA models using the empirical Bayes modal approach.^31^ Following the dementia case definition criteria in section 2.4, domain‐specific impairment was quantified as 1.5 standard deviations below the mean factor score of those aged 65 to 99 in the same education level^21^ and participants with two or more domains of cognitive impairment combined with functional impairment were defined as dementia. The same calculation process was applied to raw scores, which were the sum of original cognitive item scores for corresponding cognitive domains. The crude estimates of dementia prevalence using factor scores were then compared to that of the raw scores, and MMSE scores with education‐adjusted cut‐offs^32^ and a generic cut‐off of less than 18^33^ for dementia detection. The education‐adjusted cut‐offs were 16/17 for individual with no formal education, 19/20 for these with 1 to 6 years of education, 23/24 for these with 7 or more years of education, as suggested by a cross‐national normative study on MMSE cut‐offs.^32^ Age‐standardized dementia prevalence estimated by our latent approach was further compared to estimates reported by a nationwide survey, the COAST study^3^ and by the Global Burden of Disease (GBD) group.^34^ Dementia prevalence estimates per age group for CLHLS and CHARLS were calculated by a logistic model with age, age squared, sex, and interaction between age and sex, and adjusted for cohort‐specific survey weights. All estimates were standardized by using the 2018 population structure from the National Bureau of Statistics of China.^35^

The pairwise deletion was applied to handle missing values in CFA, and cognitive items with missing responses were replaced by zero (the lowest possible scores) for calculating the sum of the raw scores and MMSE scores (participants with five or more missing items were not included to reduce bias^36^). All analysis was conducted in R 4.0.1. Parallel analysis was conducted with the “psych” package,^37^ and CFA was performed by “lavaan” package.^38^ R Syntax for CFA and prevalence estimation were provided in supporting information.

## 3 RESULTS

### 3.1 Participant characteristics

As shown in Table 1, CHARLS participants were younger, more educated, and were less likely to have functional impairment than their CLHLS counterparts.

**TABLE 1:** Characteristics of the study samples

	**CLHLS (*N* = 12015)**	**CHARLS (*N* = 6623)**	***P*** ^a^
**Age group, *n* (%)**			<.001
65–69	1506 (12.5)	2951 (44.6)	
70–74	1713 (14.3)	1794 (27.1)	
75–79	1997 (16.6)	1106 (16.7)	
80–84	2116 (17.6)	552 (8.3)	
85–89	1675 (13.9)	176 (2.7)	
90–94	1908 (15.9)	35 (0.5)	
95–99	1100 (9.2)	9 (0.1)	
**Sex, *n* (%)**			.06
Male	6210 (51.7)	3326 (50.2)	
Female	5805 (48.3)	3297 (49.8)	
**Education level, *n* (%)**			<.001
0 years	5169 (43.0)	2090 (31.6)	
1–6 years	4331 (36.0)	3204 (48.4)	
7 years or higher	2506 (20.9)	1329 (20.1)	
*Missing*	9 (0.1)	0 (0.0)	
**Functional impairment, *n* (%)** ^b^			<.001
No	10098 (84.0)	5927 (89.5)	
Yes	1891 (15.7)	693 (10.5)	
*Missing*	26 (0.2)	3 (0.0)	

Abbreviations: CHARLS, China Health and Retirement Longitudinal Study; CLHLS, Chinese Longitudinal Healthy Longevity Survey.

^a^ *P* calculated by chi‐square test for categorical variables.

^b^ Functional impairment defined as participants who had difficulty conducting one or more activities of daily living: getting in or out of bed, bathing, dressing, cutting food, eating, using the toilet, and controlling urination and bowel movement.

### 3.2 Three‐factor CFA models for cognitive measures of CLHLS and CHARLS

**FIGURE 2:**
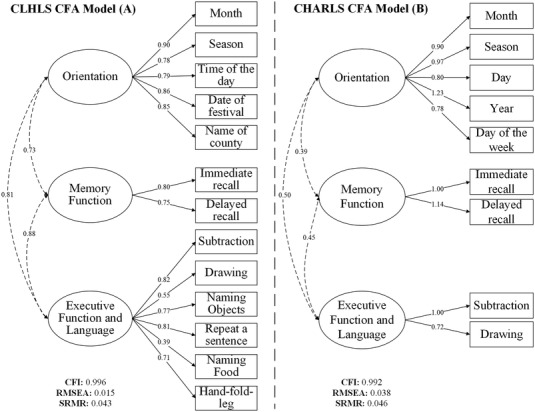
Three‐factor confirmatory factor analysis models with parameter estimates and model fit indexes for cognitive measures in CLHLS and CHARLS. CLHLS was set as the reference cohort, and mean and variance of the factor (orientation) in CLHLS were set to 0 and 1, respectively. Factor loading and threshold of the *month* item were constrained to be equivalent across cohorts, and all other factor loadings were freely estimated in reference to the fixed factor loading in CHARLS. CFA, confirmatory factor analysis; CFI, comparative fit index, acceptable value of CFI > 0.95; CHARLS, China Health and Retirement Longitudinal Study; CLHLS, Chinese Longitudinal Healthy Longevity Survey; RMSEA, root mean square error of approximation, acceptable value of RMSEA < 0.06; SRMR, standardized root mean square residual, acceptable value of SRMR < 0.08

The parallel analysis suggested three latent factors for CLHLS and CHARLS (Figure S1 in supporting information). The factors were interpreted in reference to previous cognitive literature and clinical diagnostic criteria (Table S3 in supporting information), and three‐factor CFA models with orientation, memory, plus executive function and language domains were then estimated for CLHLS and CHARLS stepwise. As shown in Figure 2, for both cohorts, all estimated factor loadings were *>*0.60 except CLHLS “naming food” 0.39 for corresponding domains with good model‐fit values for all three model‐fit indexes, indicating the common psychometric structure across cohorts. Factor scores of these three identified cognitive domains (i.e., orientation, memory, and executive function and language) were then used for dementia prevalence estimation.

### 3.3 Comparison of crude dementia prevalence using factor scores, raw scores, and MMSE scores in CLHLS and CHARLS

Table 2 shows the dementia prevalence estimates by factor scores, raw scores, and MMSE. For CLHLS, the overall estimates using factor scores were similar with MMSE estimates, and all higher than the raw score estimate. Age‐group specifically, factor score estimates tended to be higher than those of MMSE among participants younger than 75, while lower than MMSE estimates for those 85+. Corresponding estimates by raw score of those 85+ were even lower. For CHARLS, the overall and age group–specific estimates were similar between factor scores and raw scores, which were six to eight times less than corresponding estimates by MMSE with either cut‐off criteria.

**TABLE 2:** Comparison of crude dementia prevalence using factor scores, raw scores and MMSE with different cut‐offs

Cohort	Age group	Factor scores	Raw scores	MMSEeducation adjusted cut‐off	MMSEcut‐off of <18
CLHLS 2018	Overall	6.3 (5.8,6.7)	5.0 (4.6,5.4)	6.6 (6.1,7.1)	5.6 (5.2,6.0)
	65–69	3.1 (2.2,4.1)	3.0 (2.2,4.0)	1.2 (0.7,1.9)	0.4 (0.1,0.9)
	70–74	3.0 (2.2,3.9)	2.8 (2.1,3.7)	2.0 (1.4,2.8)	0.5 (0.2,1.0)
	75–79	3.5 (2.7,4.4)	3.0 (2.3,3.9)	3.3 (2.6,4.2)	2.3 (1.7,3.0)
	80–84	4.5 (3.7,5.5)	4.2 (3.4,5.2)	4.6 (3.7,5.6)	3.7 (3.0,4.7)
	85–89	6.3 (5.2,7.6)	5.0 (4.0,6.1)	9.3 (8.0,10.9)	8.6 (7.2,10.1)
	90–	12.8 (11.6,14.0)	9.1 (8.1,10.2)	15.2 (13.8,16.6)	14.0 (12.7,15.4)
CHARLS 2018	Overall	5.6 (5.0,6.1)	4.7 (4.2,5.3)	38.9 (37.6,40.1)	30.7 (29.5,31.9)
	65–69	3.6 (2.9,4.3)	3.2 (2.6,3.8)	33.8 (32.0,35.7)	26.1 (24.5,27.9)
	70–74	5.2 (4.3,6.4)	4.4 (3.5,5.5)	36.6 (34.2,39.0)	28.3 (26.1,30.6)
	75–79	6.5 (5.1,8.1)	5.2 (4.0,6.7)	45.8 (42.6,49.1)	35.5 (32.4,38.7)
	80–84	8.9 (6.6,11.6)	8.0 (5.9,10.6)	56.1 (51.2,60.9)	49.1 (44.2,53.9)
	85–89	19.9 (14.3,26.6)	15.9 (10.8,22.2)	60.0 (50.7,68.8)	55.8 (46.5,64.9)
	90–	29.5 (16.8,45.2)	27.3 (15.0,42.8)	65.2 (42.7,83.6)	52.2 (30.6,73.2)

Abbreviations: CHARLS, China Health and Retirement Longitudinal Study; CLHLS, Chinese Longitudinal Healthy Longevity Survey; MMSE, Mini‐Mental State Examination.

*Note*: Factor scores were derived from the confirmatory factor analysis models with three cognitive domains: orientation, memory, and executive function and language. Raw scores were the sum of original cognitive item scores for corresponding cognitive domains. Two cut‐off criteria were applied for MMSE score. Education‐adjusted cut‐offs were 16/17 for no formal education, 19/20 for 1 to 6 years of education, 23/24 for 7 or more years of education,^32^ and a no‐education adjusted specific cut‐off of <18.^49^

### 3.4 Age‐standardized dementia prevalence by factor scores compared to literature

**FIGURE 3:**
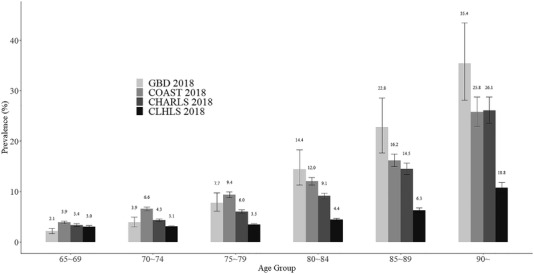
Age‐standardized dementia prevalence with 95% confidence interval using factor scores in CLHLS and CHARLS in reference to previous studies. All estimated prevalences were standardized by using the 2018 population structure from the National Bureau of Statistics of China. CHARLS, China Health and Retirement Longitudinal Study; CLHLS, Chinese Longitudinal Healthy Longevity Survey; COAST 2018, Jia et al.;^3^ GBD, the Global Burden of Disease study; GBD 2018.^50^

Figure 3 illustrates age‐standardized dementia prevalence estimated by factor scores of CLHLS and CHARLS compared to the current best evidence on dementia prevalence in China. For the age group of 65 to 69, all four estimates were close to each other, while for age groups above 70, CLHLS estimates were lower than those of CHARLS, which were in the confidence intervals of GBD and COAST estimates. It is noted that wide confidence intervals were shown for GBD estimates above 85, and CHARLS and COAST estimates above 90, indicating large uncertainties involved.

## 4 DISCUSSION

Our study aimed to identify cognitive measures of two population‐representative aging cohorts into common latent cognitive constructs to facilitate nationwide dementia prevalence estimation. We verified a common three‐factor cognitive construct with orientation, memory, and executive function and language for both cohorts with adequate model fits. Crude dementia prevalence estimated by factor scores was similar with MMSE in CLHLS, and was more reliable in CHARLS. Age‐standardized dementia estimates of CLHLS were lower than CHARLS among those aged 70+, which were close to the nationwide prevalence reported by the COAST study and GBD.

Extending previous studies that used only one latent factor^39^ or one single survey,^40^ we identified a common three‐factor CFA model suitable across two cohorts. A prior study using Health and Retirement Study sister cohorts also found that bifactor models with memory‐ and non–memory‐specific domains provided better model fit than single‐factor general cognitive function models, and memory‐specific factors should be included in latent variables analysis for CHARLS.^14^ The identification of multi‐domain cognitive constructs further facilitates the detection of dementia, where impairment in two or more cognitive domains is an essential criterion in clinical diagnosis. Our cross‐cohort comparison was guaranteed by both EFA and good model fits for CFA, suggesting configural invariance (i.e., equivalence of model form) can be confirmed for cognitive measures of both cohorts.^14^ Few prior CFA studies have investigated the factor structure of neuropsychological test variables,^41^, ^42^ and they mainly focused on methodology application while overlooking its clinical relevance or the combination with physical impairment.^39^ Our study contributed to the literature by constructing multi‐domain‐specific cognitive scores, and considering physical and cognitive function measures simultaneously, which closely resembled the DSM in detecting dementia.^18^

Moreover, dementia prevalence estimated by our latent approach tended to be more reliable than that of the raw scores and the MMSE approach across cohorts. As demonstrated in a prior methodology comparison study,^43^ the CFA approach is superior to the summary raw score, by extracting latent factor scores with unequal weighting of different cognitive tests. Compared to the MMSE estimates, factor score estimates were also less sensitive to characteristics influencing cognitive performance.^10^ Although MMSE is the most frequently used cognitive measure, it is heavily influenced by education attainment^44^ and unsuitable for individuals with low literacy.^45^ Our study found factor score estimates were closer to those of MMSE with education‐adjusted cut‐off than those with a general cut‐off for CLHLS, and were more reliable than either MMSE estimates for CHARLS. CLHLS and CHARLS both have almost 80% participants with education level less than 7 years, while only CLHLS used a modified version of MMSE.^15^ A further subgroup analysis by education level of these two cohorts indicated that these divergences in dementia prevalence estimation between factor scores and MMSE scores were most evident among participants with no formal education (see details in Table S4 in supporting information); suggesting the extremely high dementia prevalence estimates by MMSE in CHARLS may largely be attributed to the MMSE version without modification to literacy.^44^

Our latent approach was further supported by the comparison to literature on nationwide prevalence estimation. The age‐standardized dementia prevalence of CHARLS was close to that of the COAST study and GBD study, but corresponding CLHLS estimates tended to be lower than all three of them. One potential explanation is that CLHLS is designed to explore the determinants of healthy longevity in China. To fulfil this aim, CLHLS oversampled participants aged 80 years from longevity counties and cities, and the study sample is more likely to be biased by healthy participant selection. The prevalence estimated by CHARLS was close to the COAST study, a nationwide cross‐sectional dementia survey with clinical verification,^3^ suggesting that CHARLS may provide an alternative reliable data source for dementia estimates in China.

Strengths of our study include taking maximum advantages of existing population‐based surveys, with large sample sizes and high quality of data collected. We used the latent variable approach to allow estimation of cognitive performance on a common cognitive structure using all available cognitive data and accounting for measurement errors; this method is scalable with more data. Several limitations are worth notice. First, we examined configural invariance confined by the limited number of identical cognitive items across cohorts. Two cognitive items, namely, month and season, were included in both cohorts. Participants’ answers to the question on season are likely to be subjective, for example influenced by local culture, weather, and their geographical location.^46^ Therefore, we only constrained the more objective item on month. A thorough investigation on metric, scalar, and residual equivalence would be ideal to fully assess measurement invariance.^47^ Given the equivalence of latent cognitive structure is more relevant for our dementia case definition, we focused on verifying configural invariance and maintained all available cognitive tests. Second, because both surveys did not have clinical diagnoses of dementia, our estimations can only rely on cognitive test scores and functional measures collected in the datasets. Despite our dementia case definition closely resembling the clinical criteria, we acknowledged that our method required further clinical verification. Third, we addressed missing values in cognitive measures mainly assuming missing completely at random by applying WLSMV. Although alternative maximum likelihood estimation with robust standard errors (MLR) may allow more general missing at random assumption, comparable performance of WLSMV and MLR has been found with non‐normally distributed data.^48^ Given the proportion of missing values in our dataset is less than 10%, we suspected any bias due to different missing mechanisms would be small. Fourth, we only used one wave of both cohorts. This approach impedes us from identifying potential transient cognitive impairment (those who may recover in later assessments), and requires additional waves of data in the coming years to verify.

## 5 CONCLUSION

Our study identified similar cognitive constructs of two population‐representative aging cohorts in China. Configural invariance established in this study facilitated the calibration of different cognitive measures across the two aging cohorts in China, and provides a potential approach to estimate dementia prevalence across surveys and over time at the national level.

## CONFLICTS OF INTEREST

The authors have no relevant conflicts of interest to disclose. Author disclosures are available in the supporting information.

## Supporting information

SUPPORTING INFORMATION

SUPPORTING INFORMATION
