# Supplementary material for: Is there a common latent cognitive construct for dementia estimation across two Chinese cohorts?
Source: Alzheimers Dement (Amst). 2022 Sep 14;14(1):e12356. doi: 10.1002/dad2.12356 (PMC9473486; doi:10.1002/dad2.12356)
Supplement: Supplementary file 1 — SUPPORTING INFORMATION [file DAD2-14-e12356-s002.docx]

**SUPPLEMENTAL MATERIALS**

**Calibrating cognitive tests across two Chinese cohorts: a confirmatory factor analysis**

Yuyang Liu, Yanjuan Wu, Jingheng Cai, Yun Huang, Yuntao Chen, Tishya M Venkatraman, Sophia Lobanov-Rostovsky, Piotr Bandosz, Yung-Jen Yang, Yu-Tzu Wu, Jing Liao, Yuantao Hao, Eric J. Brunner

Table S1. Functional items coding and definition of FI

Table S2. Comparison between DSM-IV Criteria and definition of this study

Figure S1. Parallel analysis of the exploratory factor analysis in CLHLS and CHARLS

Table S3. Cognitive test items and reference sources for the identified latent factors

Table S4. Subgroup analysis for MMSE scored in different educational levels

R Syntax for confirmatory factor analysis and prevalence estimation

## Table S1. Functional items coding and definition of FI

| **Studies** | **Options** | **Considered as** | **Definition of FI** |
| --- | --- | --- | --- |
| CLHLS | 1. Without assistance from people. | independence | (1) Participants who were dependent on one or more ADLs as with FI.  (2) Participants who were independent in all six ADLs were defined as without FI.  (3) For those who missed some ADL items and were independent on the rest of ADLs, if they were independent in bathing or all instruments of activities in daily life (IADLs), they were defined as without FI. |
|  | 2. Need assistance partly. | dependence |  |
|  | 3. Need assistance completely. | dependence |  |
| CHARLS | 1. No, I don't have any difficulty. | independence |  |
|  | 2. I have difficulty but can still do it. | independence |  |
|  | 3. Yes, I have difficulty and need help. | dependence |  |
|  | 4. I cannot do it. | dependence |  |

FI, functional impairment

## Table S2. Comparison between DSM-IV Criteria and definition of this study

| **DSM-IV Criteria** | **CHARLS measures available** | **CLHLS measures available** |
| --- | --- | --- |
| Criterion A1 (memory impairment)  Criterion A2 ((at least one below)  a) Criterion A2a. Aphasia  b) Criterion A2b. Apraxia  c) Criterion A2c. Agnosia  d) Criterion A2d. Disturbance in executive functioning | Memory recall- three words  Orientation  Executive function & language: | Memory recall- ten words  Orientation  Executive function & language: |
|  | Cognitive impairment was defined as an impairment in two or more domains of cognitive function.  Domain impairment was defined as a factor score of 1.5 standard deviations below the mean compared with the population aged 65+ years with the same level of education | |
| Criterion B1  cause significant impairment in social or occupational functioning | ADLs consist of six items: (1) getting in or out of bed, (2) bathing, (3) dressing, (4) cutting food and eating, (5) using the toilet, and (6) controlling urination and bowel movement.  Considered to be present where the participant or the informant reported memory impairment that was hindering real-life functioning, and the informant reported one or more examples of general social/occupational impairment. | |
| Criterion B2\C\D | The available information is not enough. Besides, criterion C & D are mainly used for clinical differential diagnosis, and it is hard to tell from the epidemiological findings. | |

**A. The development of multiple cognitive deficits manifested by both**

1. memory impairment (impaired ability to learn new information or to recall previously learned information)

2. one (or more) of the following cognitive disturbances:

a. aphasia (language disturbance)

b. apraxia (impaired ability to carry out motor activities despite intact motor function)

c. agnosia (failure to recognize or identify objects despite intact sensory function)

d. disturbance in executive functioning (i.e., planning, organizing, sequencing, abstracting)

**B. The cognitive deficits in Criteria A1 and A2 each**

1. cause significant impairment in social or occupational functioning, and

2. represent a significant decline from a previous level of functioning.

**C. The deficits do not occur exclusively during the course of a delirium.**

**D. The disturbance is not better accounted for by another axis I disorder** (for example, major depressive disorder, schizophrenia)

## Figure S1. Parallel analysis of the exploratory factor analysis in CLHLS and CHARLS


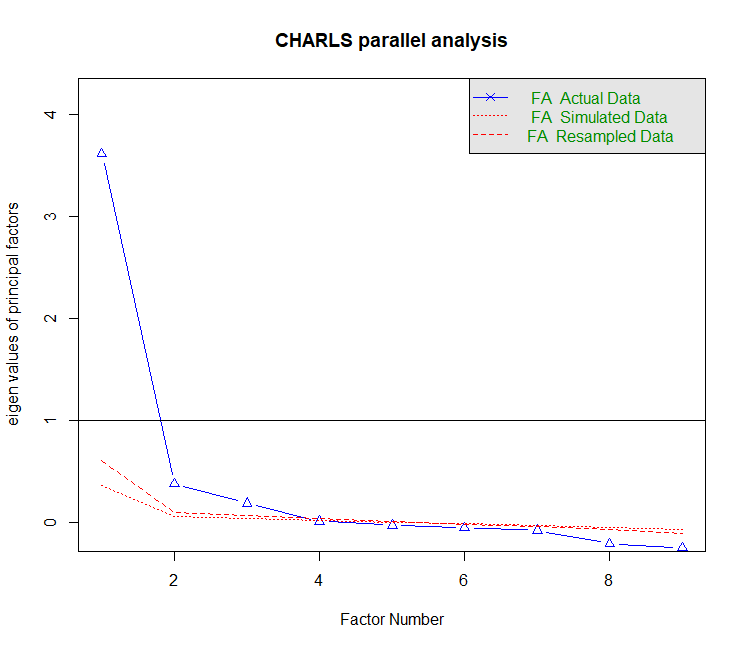

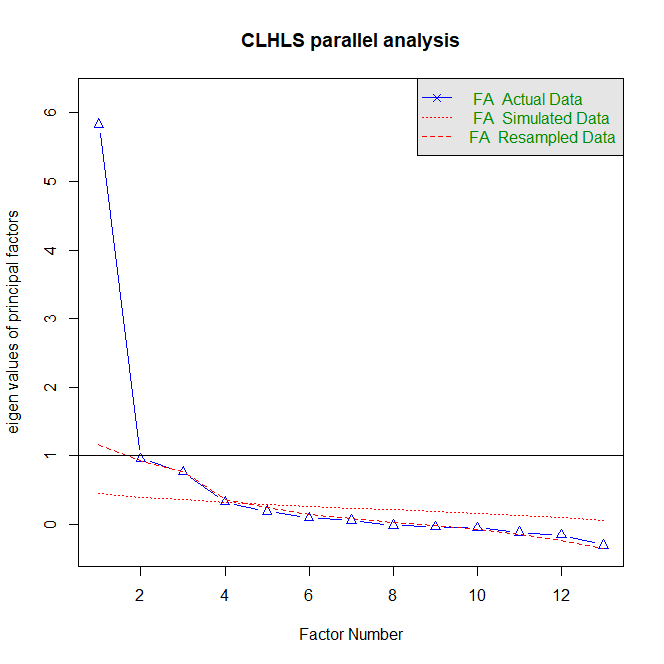


## Table S3: Cognitive test items and reference sources for the identified latent factors

| Latent Factor | Cognitive Items^*^ | | References |
| --- | --- | --- | --- |
|  | CLHLS | CHARLS |  |
| Orientation | Month | Month | Folstein (1975)  Hanzawa (1994) |
|  | Season | Season |  |
|  | Time of the day | Day |  |
|  | Date of the mid-autumn Festival | Year |  |
|  | Name of county | Day of week |  |
| Memory | 3 word:  Immediate recall  Delayed recall | 10 word:  Immediate recall  Delay recall | Folstein (1975)  Hanzawa (1994)  DSM-IV-TR |
| Executive function & Language | Repeat a sentence | / | Folstein (1975) |
|  | Food naming | / |  |
|  | Hand-fold-leg | / |  |
|  | Object naming | / |  |
|  | Drawing two vertical pentagons | Drawing two parallel pentagons | DSM-IV-TR |
|  | 20 subtract three for five times | 100 subtract seven for five times |  |

CLHLS: Chinese Longitudinal Healthy Longevity Survey; CHARLS: China Health and Retirement Longitudinal Study

* CLHLS cognitive items from the Chinese Mini-Mental State Examination (C-MMSE); CHARLS cognitive items from the Telephone Interview for Cognitive Status questionnaire (TICS)

Folstein (1975): Folstein, M. F., Folstein, S. E., & McHugh, P. R. (1975). “Mini-mental state”. Journal of Psychiatric Research, 12(3), 189-198.

Hanzawa (1994): Desmond, D. W., Tatemichi, T. K., & Hanzawa, L. (1994). The Telephone Interview for Cognitive Status (TICS): Reliability and validity in a stroke sample. International Journal of Geriatric Psychiatry, 9(10), 803-807.

DSM-IV-TR: Association, A. P. (2000). Diagnostic and Statistical Manual of Mental Disorders, Fourth Edition, Text Revision (DSM-IV-TR) (text revised ed.)

##

## Table S4. Subgroup analysis for MMSE score in different educational levels

|  | **0 year** | | **1-6 year** | | **7 years** | |
| --- | --- | --- | --- | --- | --- | --- |
|  | CHARLS | CLHLS | CHARLS | CLHLS | CHARLS | CLHLS |
| All participants, **N** | 1542 | 4708 | 2902 | 4205 | 1278 | 2472 |
| **MMSE score** |  |  |  |  |  |  |
| Mean (SD) | 15.0 (5.33) | 24.6 (4.91) | 21.2 (5.05) | 27.2 (3.64) | 24.9 (3.66) | 28.1 (3.22) |
| **Dementia, n (%)** |  |  |  |  |  |  |
| Educational Cut-Off | 938 (60.8) | 363 (7.7) | 956 (32.9) | 205 (4.9) | 329 (25.7) | 183 (7.4) |
| Cut-Off of <18 | 1037 (67.3) | 451 (9.6) | 655 (22.6) | 134 (3.2) | 63 (4.9) | 50 (2.0) |

CLHLS, Chinese Longitudinal Healthy Longevity Survey; CHARLS, China Health and Retirement Longitudinal Study; MMSE, Mini Mental State Examination.

## R Syntax for confirmatory factor analysis and prevalence estimation

# Load package ------------------------------------

library(openxlsx);

library(ggplot2);

library(readstata13)

library(psych);

library(reshape2);

library(semPlot);

# Function for standardised process --------------------

est_std_rate_ci95_new <- function(logit_data = predata,

std_data = nbsdata_0

){

std_data<-merge(nbsdata_0,logit_data,

by=c("rage", "gender"))

std_data$rage<-as.numeric(std_data$rage)

est_std_rate_ci95 <- function(index_data){

# reference

# Roalfe, A. K., Holder, R. L., & Wilson, S. (2008).

# Standardisation of rates using logistic regression:

# a comparison with the direct method. BMC Health Serv Res, 8, 275.

# doi:10.1186/1472-6963-8-275

#

# input data: age & sex-specific standard people: index_data$nb

# logistic regression: logit: index_data$logit

index_data$a<-index_data$nbs*index_data$logit

b<-sum(index_data$a[1:nrow(index_data)])

N<-sum(index_data$nbs[1:nrow(index_data)])

std_logit<-b/N

std_rate<-exp(std_logit)/(1+exp(std_logit))

index_data$m<-((index_data$se_logit)^2)*((index_data$nbs)^2)

n<-sum(index_data$m[1:nrow(index_data)])

se_std_logit<-sqrt(n)/N

upper<-std_logit+1.96*se_std_logit

lower<-std_logit-1.96*se_std_logit

std_rate_upper<-exp(upper)/(1+exp(upper))

std_rate_lower<-exp(lower)/(1+exp(lower))

data_result <- data.frame(rate = std_rate,upper = std_rate_upper,

lower = std_rate_lower)

result<-paste(round(100*std_rate,1),"(",

round(100*std_rate_lower,1),",",

round(100*std_rate_upper,1),")")

return(list(data_result=data_result,result =result))

}

std_data$agecat<-

ifelse(std_data$rage<70,1,

ifelse(std_data$rage<75,2,

ifelse(std_data$rage<80,3,

ifelse(std_data$rage<85,4,

ifelse(std_data$rage<90,5,

ifelse(std_data$rage<95,6,7))))))

std_data_m<-subset(std_data,gender==1)

std_data_f<-subset(std_data,gender==0)

dementia_pre<-data.frame()

dementia_pre.data<-data.frame(agegroup = NA)

for (i in 1:7){

a<- subset(std_data,agecat==i)

dementia_pre[i+1,1]<-est_std_rate_ci95(a)$result

dementia_pre.data[i+1,'agegroup'] <- i

dementia_pre.data[i+1,c('rate ','upper','lower' )] <-

est_std_rate_ci95(a)$data_result

}

# Overall, regardless of age group

dementia_pre[1,1]<-est_std_rate_ci95(std_data)$result

dementia_pre.data[1,c('agegroup' )]<- 'Overall'

dementia_pre.data[1,c('rate ','upper','lower' )]<-

est_std_rate_ci95(std_data)$data_result

names(dementia_pre)<-c("overall","male","female")

return(list(dementia_pre = dementia_pre,dementia_pre.data = dementia_pre.data))

}

# Predict data.frame

predata0 <- data.frame(rage = 65:99, gender = 0)

predata1 <- data.frame(rage = 65:99, gender = 1)

predata <- rbind(predata0,predata1)

#_____________________________________________________________----

# Prevalence of Factor scores --------------------------------

## CFA Model -------------------------------------------

clhls_cfa_model <- "

Factor_1 =~ NA*imrcr_f + dlrcr_f

Factor_2 =~ NA*ser_f + mor_f + ctr_f + dtr_f + atr_f

Factor_3 =~ NA*nmr_f + drawr_f + exr_f + sersr_f + vbrcut_f + reptr_f

#variance

Factor_2 ~~ 1*Factor_2

Factor_1 ~~ 1*Factor_1

Factor_3 ~~ 1*Factor_3

#mean

Factor_1 ~ 0*1

Factor_2 ~ 0*1

Factor_3 ~ 0*1

"

charls_cfa_model <- "

Factor_1 =~ 1*imrctr_f + dlrctr_f

Factor_2 =~ NA*ser_f + 0.898*mor_f + dwr_f + dyr_f + yrr_f

Factor_3 =~ 1*sersr_f + drawr_f

# threshold

mor_f | -1.599*t1

#variance

Factor_2 ~~ NA*Factor_2

#mean

Factor_2 ~ NA*1

"

## CFA construct -------------------------------------------

clhls_cfa_model_run <- cfa(clhls_cfa_model,

data = clhlsb, # CLHLS data

ordered = TRUE,

missing = "pairwise",

estimator = "WLSMV")

charls_cfa_model_run <- cfa(charls_cfa_model,

data = charlsb, , # CHARLS data

missing = "pairwise",

ordered = TRUE,

estimator = "WLSMV")

clhls_cfa_model_fit <- summary(clhls_cfa_model_run,

fit.measures=TRUE,

standardized = TRUE,rsquare=TRUE)

charls_cfa_model_fit <- summary(charls_cfa_model_run,

fit.measures=TRUE,

standardized = TRUE)

## Calculate factor score------------------

clhls_index <- lavInspect(clhls_cfa_model_run , "case.idx")

clhls_fs <- lavPredict(clhls_cfa_model_run,

method = "EBM")

for (fs in colnames(clhls_fs)) {

clhlsb[clhls_index , fs] <-

clhls_fs [ , fs]

}

charls_index <- lavInspect(charls_cfa_model_run , "case.idx")

charls_fs <- lavPredict(charls_cfa_model_run,

method = "EBM")

for (fs in colnames(charls_fs)) {

charlsb[charls_index , fs] <-

charls_fs [ , fs]

}

### Calculate 1.5SD of factor score------------------

clhlsb$f1_15sd <- NA

clhlsb$f2_15sd <- NA

clhlsb$f3_15sd <- NA

for (row in 1:nrow(clhlsb)){

clhlsb$f1_15sd[row] <- quantile(clhlsb$Factor_1[which(

clhlsb$mmsedu == clhlsb$mmsedu[row])],

0.07,na.rm = TRUE)

clhlsb$f2_15sd[row] <- quantile(clhlsb$Factor_2[which(

clhlsb$mmsedu == clhlsb$mmsedu[row])],

0.07,na.rm = TRUE)

clhlsb$f3_15sd[row] <- quantile(clhlsb$Factor_3[which(

clhlsb$mmsedu == clhlsb$mmsedu[row])],

0.07,na.rm = TRUE)

}

for (row in 1:nrow(charlsb)){

charlsb$f1_15sd[row] <- quantile(charlsb$Factor_1[which(

charlsb$mmsedu == charlsb$mmsedu[row])],

0.07,na.rm = TRUE)

charlsb$f2_15sd[row] <- quantile(charlsb$Factor_2[which(

charlsb$mmsedu == charlsb$mmsedu[row])],

0.07,na.rm = TRUE)

charlsb$f3_15sd[row] <- quantile(charlsb$Factor_3[which(

charlsb$mmsedu == charlsb$mmsedu[row])],

0.07,na.rm = TRUE)

}

### Define domain impairment ------------------------

clhlsb$f1_ci <- ifelse(clhlsb$Factor_1 < clhlsb$f1_15sd,1,0)

clhlsb$f2_ci <- ifelse(clhlsb$Factor_2 <clhlsb$f2_15sd,1,0)

clhlsb$f3_ci<- ifelse(clhlsb$Factor_3 <clhlsb$f3_15sd,1,0)

charlsb$f1_ci <- ifelse(charlsb$Factor_1 < charlsb$f1_15sd,1,0)

charlsb$f2_ci <- ifelse(charlsb$Factor_2 <charlsb$f2_15sd,1,0)

charlsb$f3_ci<- ifelse(charlsb$Factor_3 <charlsb$f3_15sd,1,0)

### Define cognitive impairment ------------------------

for (i in 1:nrow(clhlsb)){#CLHLS

if (is.na(clhlsb$f1_ci[i]) == FALSE&

is.na(clhlsb$f2_ci[i]) == FALSE&

is.na(clhlsb$f3_ci[i]) == FALSE){

clhlsb$f_ci[i] <- ifelse(sum(clhlsb$f1_ci[i],

clhlsb$f2_ci[i],

clhlsb$f3_ci[i],na.rm = TRUE) >=2,1,0)

}

}

for (i in 1:nrow(charlsb)){

if (is.na(charlsb$f1_ci[i]) == FALSE&

is.na(charlsb$f2_ci[i]) == FALSE&

is.na(charlsb$f3_ci[i]) == FALSE){

charlsb$f_ci[i] <- ifelse(sum(charlsb$f1_ci[i],

charlsb$f2_ci[i],

charlsb$f3_ci[i],na.rm = TRUE) >=2,1,0)

}

}

### Merge data -----------------------

clhls_all<-merge(clhls,clhlsb[,c("ID","f_ci")],

by=c("ID"),

all= TRUE)

charls_all<-merge(charls,charlsb[,c("ID","f_ci")],

by=c("ID"),

all= TRUE)

### Dementia definetion -------------------------

clhls_all$f_dementia<- NA

for (r in 1:nrow(clhls_all)){

if (clhls_all[r,"f_ci"] == 1 &

clhls_all[r,"adlsixrf"] == 1 &

is.na(clhls_all[r,"f_ci"]) == FALSE &

is.na(clhls_all[r,"adlsixrf"]) == FALSE ){

# adlsixrf is ADL information(0 means Non-FI, 1 means FI)

clhls_all$f_dementia[r] <- 1

}else if(clhls_all$demntia[r]==1&

is.na(clhls_all$demntia[r]) == FALSE){

# demntia is self-reported doctor-diagnosed dementia

clhls_all$f_dementia[r]<-1

}else{

clhls_all$f_dementia[r] <- 0

}

}

charls_all$f_dementia<- NA

for (r in 1:nrow(charls_all)){

if (

charls_all[r,"memryer"] == 1 &

is.na(charls_all[r,"memryer"]) == FALSE ){

# memryer is self-reported doctor-diagnosed memory-related diseases

charls_all$f_dementia[r] <- 1

}else if(charls_all[r,"f_ci"] == 1 &

charls_all[r,"adlsixrf"] == 1 &

is.na(charls_all[r,"f_ci"]) == FALSE &

is.na(charls_all[r,"adlsixrf"]) == FALSE) {

# adlsixrf is ADL information(0 means Non-FI, 1 means FI)

charls_all$f_dementia[r] <- 1

}else{

charls_all$f_dementia[r] <- 0

}

}

### Observation Prevalence---------------------------------

obs_fac <- rbind(data.frame(rbind(table(clhls_all$f_dementia),

table(clhls_all$agecat,clhls_all$f_dementia)),

agegroup = c('Overall','65~69','70~74','75~79','80~84',

'85~89','90~94','95~99'),

data = 'CLHLS',

type = 'Factor scores'),

data.frame(rbind(table(charls_all$f_dementia),

table(charls_all$agecat,charls_all$f_dementia)),

agegroup = c('Overall','65~69','70~74','75~79','80~84',

'85~89','90~94','95~99'),

data = 'CHARLS',

type = 'Factor scores'))

for (r in 1:nrow(obs_fac)){

result_obsfac <- binom.test(x = (obs_fac[r,2]),

n = (obs_fac[r,1] + obs_fac[r,2]),

p = (obs_fac[r,2]/(obs_fac[r,1] + obs_fac[r,2])),

conf.level = 0.95)

obs_fac$estrate[r] <- result_obsfac$estimate

obs_fac$up[r] <- result_obsfac$conf.int[2]

obs_fac$down[r] <- result_obsfac$conf.int[1]

obs_fac$result[r] <- paste0(sprintf('%.1f',obs_fac$estrate[r]*100),' (',

sprintf('%.1f',obs_fac$down[r]*100),',',

sprintf('%.1f',obs_fac$up[r]*100),')')

}

###logistic model-----

clhls_all$gender <- ifelse(clhls_all$gender == 1,1,0)

charls_all$gender <- ifelse(charls_all$gender == 1,1,0)

#### Standardized Process ------------------------------------------------------------------

clhls_dem = glm(f_dementia ~rage + I(rage^2)+gender + rage*gender,

# family = binomial("logit"),

family = quasibinomial("logit"),

weights = weight,

data = clhls_all)

charls_dem = glm(f_dementia ~rage + I(rage^2)+gender + rage*gender,

# family = binomial("logit"),

family = quasibinomial("logit"),

weights = weight/1000000,

data = charls_all)

## standardization & confidence interval-----

### CLHLS-dementia-----

predata_clhls <- predata

predata_clhls$logit<- predict(clhls_dem,predata_clhls,

se.fit = TRUE)$fit

predata_clhls$se_logit<-predict(clhls_dem,predata_clhls,

se.fit = TRUE)$se.fit

clhls_dementia_pre <- est_std_rate_ci95_new(logit_data = predata_clhls,

std_data = nbsdata_0)

### CHARLS-dementia-----

predata_charls <- predata

predata_charls$logit<- predict(charls_dem,predata_charls,

se.fit = TRUE)$fit

predata_charls$se_logit<-predict(charls_dem,predata_charls,

se.fit = TRUE)$se.fit

charls_dementia_pre <- est_std_rate_ci95_new(logit_data = predata_charls,

std_data = nbsdata_0)

# Standardized Prevalence

table23fac <- list(clhls_dementia_pre,

charls_dementia_pre )
